# Sex differences in the progression of cerebral microbleeds in patients with concomitant cerebral small vessel disease

**DOI:** 10.3389/fneur.2022.1054624

**Published:** 2022-12-20

**Authors:** Cindy W. Yoon, Joung-Ho Rha, Hee-Kwon Park, Soo-Hyun Park, Soonwook Kwon, Byeong C. Kim, Young Chul Youn, Jee Hyang Jeong, Hyun Jeong Han, Seong Hye Choi

**Affiliations:** ^1^Department of Neurology, Inha University School of Medicine, Incheon, Republic of Korea; ^2^Department of Neurology, Chonnam National University Medical School, Gwangju, Republic of Korea; ^3^Department of Neurology, Chung-Ang University College of Medicine, Seoul, Republic of Korea; ^4^Department of Neurology, Ewha Womans University School of Medicine, Seoul, Republic of Korea; ^5^Department of Neurology, Myongji Hospital, Hanyang University College of Medicine, Goyang, Republic of Korea

**Keywords:** sex differences, women, microbleeds, cerebral microbleeds, cerebral small vessel disease

## Abstract

**Background and purpose:**

Sex differences in cerebral microbleeds (CMBs) are not well-known. We aimed to assess the impact of sex on the progression of CMBs.

**Methods:**

The CHALLENGE (Comparison Study of Cilostazol and Aspirin on Changes in Volume of Cerebral Small Vessel Disease White Matter Changes) database was analyzed. Out of 256 subjects, 189 participants with a follow-up brain scan were included in the analysis. The linear mixed-effect model was used to compare the 2-year changes in the number of CMBs between men and women.

**Results:**

A total of 65 men and 124 women were analyzed. There were no significant differences in the prevalence (70.8 vs. 71.8%; *P* = 1.000) and the median [interquartile range (IQR)] number of total CMBs [1 (0–7) vs. 2 (0–7); *P* = 0.810] at baseline between men and women. The median (IQR) increase over 2 years in the number of CMBs was statistically higher in women than in men [1 (0–2) vs. 0 (0–1), *P* = 0.026]. The multivariate linear mixed-effects model showed that women had a significantly greater increase in the number of total, deep, and lobar CMBs compared to men after adjusting for age and the baseline number of CMBs [estimated log-transformed mean of difference between women and men: 0.040 (*P* = 0.028) for total CMBs, 0.037 (*P* = 0.047) for deep CMBs, and 0.047 (*P* = 0.009) for lobar CMBs].

**Conclusion:**

The progression of CMB over 2 years was significantly greater in women than in men.

## Introduction

Sex differences have been reported in various aspects of cerebrovascular disease (CVD) (1–4). Accumulating data on CVD-related sex differences can improve understanding and planning of sex-specific care. Cerebral small vessel disease (cSVD) is one of the most common forms of CVD and is associated with stroke and dementia (5). In cSVD, sex differences can also be expected, and some relevant studies have been published (6–9). Previous studies have shown that white matter hyperintensities (WMH), one of the representative markers of cSVD, is associated with sex differences. For instance, women tend to have larger volumes and faster progression of WMH compared to men (6–9).

Cerebral microbleed (CMB) is also considered one of the cSVD markers (10, 11). CMB is a clinically important marker of a bleeding-prone microangiopathy that is associated with hemorrhagic stroke and hemorrhagic complication following antithrombotic therapy (12). CMBs are also known to be associated with risks of cognitive decline and dementia (13). However, sex differences in CMBs are not well-known. In this study, we aimed to explore the impact of sex on the progression of CMBs using longitudinal data from the CHALLENGE (Comparison Study of Cilostazol and Aspirin on Changes in Volume of Cerebral Small Vessel Disease White Matter Changes) trial (14).

## Methods

### Study participants

This study is a sub-analysis of the CHALLENGE (Clinicaltrials.gov; Unique identifier: NCT01932203) trial, a multicenter, double-blind, randomized controlled trial that enrolled participants aged 50–85 years with cSVD (14). The diagnosis of cSVD was established based on the presence of at least one lacune and moderate to severe WMH, according to the modified Fazekas criteria for periventricular WMH with a cap or rim of ≥5 mm and deep WMH with a maximum diameter of ≥10 mm (15). The main objective of the trial was to compare the effects of cilostazol and aspirin on the changes in WMH volume over 2 years. Between July 2013 and August 2016, 282 participants were screened for eligibility, of whom 256 were randomly assigned to the cilostazol or aspirin group. Out of 256 CHALLENGE subjects, 189 participants with a follow-up magnetic resonance imaging (MRI) scan were included in our analysis. The comparison between subjects with and without a follow-up MRI scan is shown in Supplementary Table 1. There were no significant differences between the two groups, including age, sex, vascular risk factors and baseline CMBs.

The Institutional Review Boards of the participating centers approved this study. The approval number of the affiliated center of the corresponding author (SC) was 2013-03-006. Written informed consent was obtained from all potential participants prior to enrollment.

### Imaging markers

Brain MRI data including axial T2^*^-weighted gradient-echo sequence (4-mm slice thickness with no interslice gap) were acquired using a 3.0 Tesla MR scanner. The same scanner and the same sequence were used for the baseline and the follow-up MRI.

CMBs were defined as lesions with a diameter of ≤ 10 mm and rated using the Microbleed Anatomical Rating Scale (MARS) (16). Two experienced neurologists, blinded to clinical information, counted the number of CMBs on gradient-echo MRI images. The Pearson's correlation coefficient of agreement on the number of CMBs between the two neurologists was 0.958 (95% confidence interval 0.809–0.989; *P* < 0.001). The two neurologists reached a consensus after discussion in cases with initial disagreement. CMBs were categorized as deep (basal ganglia, thalamus, internal/external capsule, corpus callosum, deep/periventricular white matter, and brainstem) and lobar (frontal, parietal, temporal, occipital, and insular cortices).

### Statistical analysis

The baseline characteristics were compared between men and women using the chi-square test for categorical variables and the Student's *t*-test or the Mann–Whitney *U*-test for continuous variables. The change in the number of CMBs and the proportion of patients with CMB progression (defined as an increase in the number of CMBs ≥1) during the 2-year follow-up period were compared using the Mann–Whitney *U*-test and the chi-square test.

We used the linear mixed-effects model with a random subject effect to estimate and compare changes in the number of CMBs over 2 years. To assess the trend in each group, linear mixed-model analyses were performed separately using time (baseline and 2-year follow-up visit) as a predictor. To determine the impact of sex on the longitudinal changes in CMB counts, we explored the interaction between sex and time (sex × time) adjusted for age and the number of CMBs at baseline (model 1). Model 2 was adjusted for the variables in model 1 and hypertension (HTN), diabetes, dyslipidemia, current smoking, body mass index (BMI), apolipoproteins E4 (APOE4) and E2 (APOE2), and antiplatelet medication (aspirin vs. cilostazol). The number of CMBs was logarithmically transformed due to a skewed distribution. Before the logarithmic transformation, a constant of 1 was added to all values to overcome the problem of zero values.

All statistical analyses were performed using SPSS version 23.0 (IBM SPSS Inc., USA). Two-tailed *P-*values were reported, and *P* < 0.05 was considered statistically significant.

## Results

A total of 65 men and 124 women were included in the analysis. The baseline characteristics of men and women are shown in Table 1. Compared to men, women were older: the mean age for women and men was 74.5 and 71.5 years, respectively. Men were more likely than women to be cigarette smokers at present (15.4 vs. 1.6%; *P* < 0.001). There were no significant differences between the two groups in other vascular risk factors, the proportion of APOE4 and APOE2 carriers, the antiplatelet medications administered (aspirin vs. cilostazol), and follow up duration. Regarding cSVD markers at baseline, men had a higher median [interquartile range (IQR)] number of lacunes than women [8 (4–13) vs. 5 (2–9); *P* = 0.014]. There were no significant differences in the total WMH volume, as well as in the prevalence (70.8 vs. 71.8%; *P* = 1.000) or median (IQR) number of CMBs [1 (0–7) vs. 2 (0–7); *P* = 0.810] at baseline.

**Table 1:** Comparison of baseline characteristics between men and women.

	**Men** ** (*n* = 65)**	**Women (*n* = 124)**	***P-*value**
Age, years	71.5 (7.8)	74.5 (5.9)	0.014
Hypertension	52 (80.0%)	104 (83.9%)	0.548
Diabetes	27 (41.5%)	46 (37.1%)	0.637
Dyslipidemia	33 (50.8%)	60 (48.4%)	0.759
Current Smoking	10 (15.4%)	2 (1.6%)	< 0.001
Body mass index, kg/m^2^	24.2 (2.7)	25.0 (3.2)	0.085
Systolic blood pressure, mmHg	130.8 (14.6)	129.3 (12.6)	0.464
Diastolic blood pressure, mmHg	73.8 (9.9)	73.8 (9.5)	0.986
Apolipoprotein E4 carrier	19 (29.2%)	30 (24.2%)	0.602
Apolipoprotein E2 carrier	6 (9.2%)	13 (10.5%)	0.804
Antiplatelet medication			0.542
Aspirin	33 (50.8%)	69 (55.6%)	
Cilostazol	32 (49.2%)	55 (44.4%)	
Follow-up, years	1.99 (1.98–2.02)	2.00 (1.98–2.02)	0.998
**Baseline cSVD markers**			
WMH volume, mL	34.5 (20.0–47.7)	34.8 (26.5–50.0)	0.262
Number of lacunes	8 (4–13)	5 (2–9)	0.014
Presence of CMBs	46 (70.8%)	89 (71.8%)	1.000
Number of CMBs			
Deep	1 (0–4)	1 (0–4.5)	0.291
Lobar	1 (0–3)	0 (0–2)	0.407
Total	1 (0–7)	2 (0–7)	0.810

The values are presented as percentages (%), mean (SD), or median (IQR). CMB, cerebral microbleed; cSVD, cerebral small vessel disease; IQR, interquartile range; SD, standard deviation; WMH, white matter hyperintensities.

**Figure 1:**
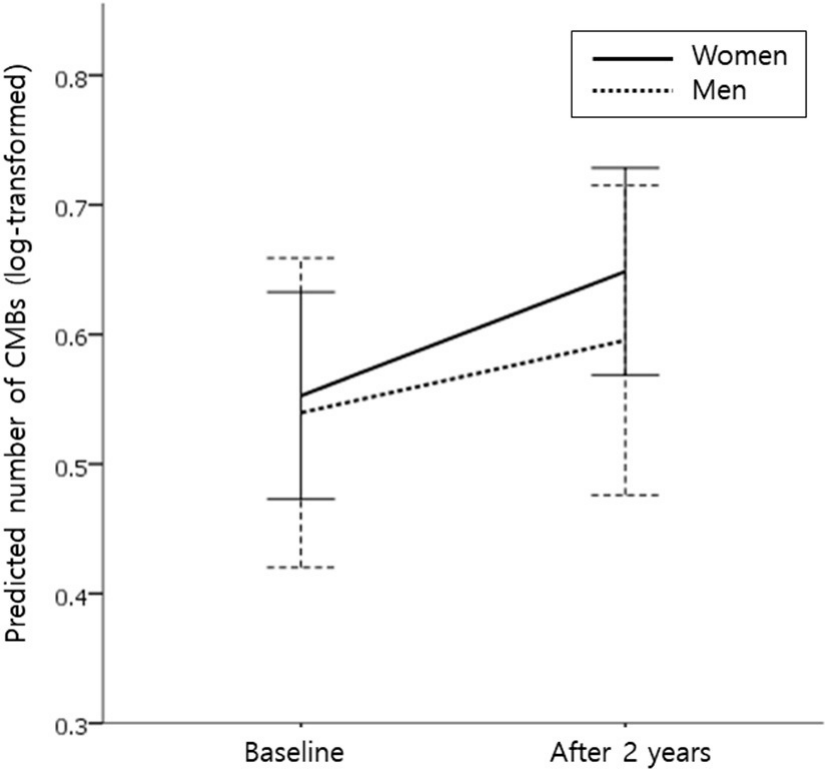
Effect of sex on longitudinal changes in the number of cerebral microbleeds (CMBs) during the 2-year follow-up period. Predicted mean values for the number of CMBs (log-transformed for normality) in women (solid line) and men (dotted line) from the linear mixed-effects model. Analysis controlled for age and the baseline number of CMBs. Error bars indicate a 95% confidence interval.

After 2 years of follow-up, the proportion of patients with CMB progression (defined as an increase in the number of CMBs by ≥ 1) tended to be higher in women than in men [54.8% (68/124) vs. 40.0% (26/65); *P* = 0.066]. The median (IQR) increase in the number of CMBs over 2 years was statistically higher in women than in men [1 (0–2) vs. 0 (0–1), *P* = 0.026]. In the linear mixed-effect model, which tested the sex x time interaction effect on changes in CMB counts, women had a much greater increase in CMB counts than men after adjusting for age and the baseline number of CMBs (model 1), regardless of the location of CMBs (deep and lobar; Table 2). Model 2, in which HTN, diabetes, dyslipidemia, current smoking, BMI, APOE4, APOE2, and antiplatelet medications were adjusted in addition to model 1 variables, showed the same result (Table 2). Figure 1 shows the estimated effect of sex on longitudinal changes in the number of CMBs over a 2-year follow-up period.

**Table 2:** Comparison of longitudinal changes in the number of cerebral microbleeds between men and women.

		**Changes in the number of CMBs over the 2-year follow-up period (log-transformed)**			
		**Men**	**Women**	**Differences between women and men** **(men as a reference)**	
		**Estimated mean (SE)**	**Estimated mean (SE)**	**Estimated mean (SE)**	***P-*value**
Total	Model 1^*^	0.056 (0.013)	0.096 (0.011)	0.040 (0.018)	0.028
	Model 2^†^	0.056 (0.013)	0.095 (0.012)	0.039 (0.018)	0.036
Deep	Model 1^*^	0.022 (0.015)	0.059 (0.011)	0.037 (0.018)	0.047
	Model 2^†^	0.022 (0.016)	0.062 (0.011)	0.040 (0.019)	0.038
Lobar	Model 1^*^	0.034 (0.013)	0.082 (0.011)	0.047 (0.018)	0.009
	Model 2^†^	0.034 (0.013)	0.080 (0.011)	0.046 (0.018)	0.012

CMBs, cerebral microbleeds; SE, Standard error.

* Results of a linear mixed model adjusted for the baseline number of CMBs and age.

† Results of a linear mixed model adjusted for the baseline number of CMBs, age, hypertension, diabetes, dyslipidemia, current smoking, body mass index, apolipoprotein E4 and E2, and antiplatelet agents (cilostazol vs. aspirin).

## Discussion

In this longitudinal study, we compared the progression of CMBs in men and women. When interpreting the results of our study, it is important to remember that our sample consisted of fairly old individuals with relatively severe pre-existing cSVD. For this reason, the proportion of patients with baseline CMB was much higher in our study (over 70% in both men and women) compared to studies that enrolled participants from the general population (17, 18). It is well-known that the baseline burden of cSVD is closely related to the progression of cSVD (19). Therefore, the progression of CMBs in our patient sample may be higher than that in the general population.

Our major finding was that women showed a much greater increase in CMB counts than men, although there was no significant difference in the degree of CMBs at baseline. This difference between the sexes persisted even after adjusting for the baseline burden and various risk factors. The cause behind the more pronounced CMB progression in women than in men is unclear; however, there are some possible explanations. The formation of CMBs is considered a microangiopathic process. Structural weakening and endothelial dysfunction of the microvasculature as a result of oxidative stress, inflammation, and degenerative changes, increased pulse pressure on the microvasculature due to arterial stiffening, as well as impaired myogenic autoregulatory protection of the cerebral microvasculature are thought to be the potential mechanisms of CMB formation (20, 21). Previous studies have demonstrated sex differences in the structure and function of blood vessels, including myogenic reactivity and endothelial function mainly by the protective role of female sex hormone, particularly estrogen (22, 23). However, postmenopausal women may be more vulnerable to CMB formation due to reduced protective effects of estrogen. Regarding WMH, another representative marker of cSVD along with CMB, previous studies have also shown that elderly women have a faster progression of WMH compared to elderly men (9, 19). It might be explained in a similar fashion that generally women have a lower prevalence of cardiovascular disease than men before menopause, however, after menopause, this trend is reversed (24). The age range of women in our study was 56–85 years, and it was very likely that these patients were in menopause. The results could be different if the comparative analysis is conducted in younger patients.

In our study, women showed a more pronounced increase in both deep and lobar CMBs. The location and distribution of CMBs are considered to reflect their underlying pathology (25). Cerebrovascular risk factors, including HTN, mainly cause deep CMBs, which reflects the involvement of deep perforating arteries (25). For lobar CMBs, the etiology is deemed similar to that of deep CMBs but only in cases of mixed CMBs, where lobar CMBs co-occur with deep CMBs (11, 26, 27). On the other hand, cases of strictly lobar CMBs are considered to be caused by cerebral amyloid angiopathy (CAA), in which amyloid deposition occurs in cortical/leptomeningeal vessels (28). We speculated that most CMBs in our patients were likely caused by vascular risk factors rather than CAA, since we included only patients with underlying lacunes and moderate to severe deep WMHs and our patients had high prevalence of HTN over 80%. Furthermore, none of our patients met the Boston criteria as possible or probable CAA (29). However, the possibility of concomitant CAA in our patients cannot be completely ruled out. In animal models of CAA, female mice had a significantly higher burden of CMBs than males (30). Further research and data collection, including those on amyloid positivity, are needed to identify sex differences in the progression of CMBs based on their underlying pathology.

The limitations of our study should be noted. The CHALLENGE trial, which formed the foundation for our analysis, was not designed to explore the differences between men and women, and there was a difference in the proportion of men and women initially enrolled in the trial. Although age and current smoking were adjusted for in the multivariate analysis, women were older than men and men were more likely to smoke than women in our study. Our patient group is not representative of the general population. As mentioned earlier, there is a possibility that the results may differ in younger people who have less burden of underlying cSVD and vascular risk factors. It should also be noted that all of our patients were taking antiplatelet agents (aspirin or cilostazol) during the follow-up period. Finally, the overall sample size of this study was relatively small. A study with a larger sample size and a more extended follow-up period may be needed to validate our findings.

## Conclusion

In this prospective study, women showed a much greater increase in CMB counts over 2 years than men. Our study highlights sex differences in the progression of CMBs, and understanding these sex differences may inform the development of sex-specific care strategies.

## Data availability statement

The datasets generated for this study will be made available on request to the corresponding author.

## Ethics statement

The studies involving human participants were reviewed and approved by the Institutional Review Board of Inha University Hospital. The patients/participants provided their written informed consent to participate in this study.

## Author contributions

CY and SC contributed to the conception and design of the study and performed the statistical analysis. CY, J-HR, H-KP, BK, YY, JJ, HH, and SC collected the data. CY, S-HP, SK, BK, YY, JJ, HH, and SC organized the database. CY wrote the first draft of the manuscript. SC reviewed and edited the manuscript. All authors have read and approved the final manuscript.

## References

[1] Bushnell CD Chaturvedi S Gage KR Herson PS Hurn PD Jiménez MC. Sex differences in stroke: challenges and opportunities. J Cereb Blood Flow Metab. (2018) 38:2179–91. 10.1177/0271678X18793324 30114967 PMC6282222

[2] Rexrode KM Madsen TE Yu AY Carcel C Lichtman JH Miller EC. The impact of sex and gender on stroke. Circ Res. (2022) 130:512–28. 10.1161/CIRCRESAHA.121.319915 35175851 PMC8890686

[3] Gao Z Chen Z Sun A Deng X. Gender differences in cardiovascular disease. Med Novel Technol Dev. (2019) 4:100025. 10.1016/j.medntd.2019.100025

[4] Robison LS Gannon OJ Salinero AE Zuloaga KL. Contributions of sex to cerebrovascular function and pathology. Brain Res. (2019) 1710:43–60. 10.1016/j.brainres.2018.12.030 30580011

[5] Wardlaw JM Smith C Dichgans M. Small vessel disease: mechanisms and clinical implications. Lancet Neurol. (2019) 18:684–96. 10.1016/S1474-4422(19)30079-1 31097385

[6] De Leeuw F de Groot JC Achten E Oudkerk M Ramos L Heijboer R. Prevalence of cerebral white matter lesions in elderly people: a population based magnetic resonance imaging study. J Neurol Neurosurg Psychiatr. (2001) 70:9–14. 10.1136/jnnp.70.1.9 11118240 PMC1763449

[7] Fatemi F Kantarci K Graff-Radford J Preboske GM Weigand SD Przybelski SA. Sex differences in cerebrovascular pathologies on FLAIR in cognitively unimpaired elderly. Neurology. (2018) 90:e466–e73. 10.1212/WNL.0000000000004913 29343465 PMC5818016

[8] Sachdev PS Parslow R Wen W Anstey K Easteal S. Sex differences in the causes and consequences of white matter hyperintensities. Neurobiol Aging. (2009) 30:946–56. 10.1016/j.neurobiolaging.2007.08.023 17950492

[9] Van Den Heuvel D Admiraal-Behloul F Ten Dam V Olofsen H Bollen E Murray H. Different progression rates for deep white matter hyperintensities in elderly men and women. Neurology. (2004) 63:1699–701. 10.1212/01.WNL.0000143058.40388.44 15534259

[10] Pasi M Cordonnier C. Clinical relevance of cerebral small vessel diseases. Stroke. (2020) 51:47–53. 10.1161/STROKEAHA.119.024148 31752613

[11] Park JH Seo SW Kim C Kim GH Noh HJ Kim ST. Pathogenesis of cerebral microbleeds: *in vivo* imaging of amyloid and subcortical ischemic small vessel disease in 226 individuals with cognitive impairment. Ann Neurol. (2013) 73:584–93. 10.1002/ana.23845 23495089

[12] Kim BJ Lee S-H. Cerebral microbleeds: their associated factors, radiologic findings, and clinical implications. J Stroke. (2013) 15:153. 10.5853/jos.2013.15.3.153 24396809 PMC3859003

[13] Akoudad S Wolters FJ Viswanathan A de Bruijn RF Van der Lugt A Hofman A. Association of cerebral microbleeds with cognitive decline and dementia. JAMA Neurol. (2016) 73:934–43. 10.1001/jamaneurol.2016.1017 27271785 PMC5966721

[14] Kim BC Youn YC Jeong JH Han HJ Kim JH Lee J-H. Cilostazol versus aspirin on white matter changes in cerebral small vessel disease: a randomized controlled trial. Stroke. (2022) 29:698–709. 10.1161/STROKEAHA.121.035766 34781708

[15] Noh Y Lee Y Seo SW Jeong JH Choi SH Back JH. A new classification system for ischemia using a combination of deep and periventricular white matter hyperintensities. J Stroke Cereb Dis. (2014) 23:636–42. 10.1016/j.jstrokecerebrovasdis.2013.06.002 23867045

[16] Gregoire S Chaudhary U Brown M Yousry T Kallis C Jäger H. The microbleed anatomical rating scale (MARS): reliability of a tool to map brain microbleeds. Neurology. (2009) 73:1759–66. 10.1212/WNL.0b013e3181c34a7d 19933977

[17] Poels MM Vernooij MW Ikram MA Hofman A Krestin GP van der Lugt A. Prevalence and risk factors of cerebral microbleeds: an update of the Rotterdam scan study. Stroke. (2010) 41:S103–S6. 10.1161/STROKEAHA.110.595181 20876479

[18] Yates PA Villemagne VL Ellis KA Desmond PM Masters CL Rowe CC. Cerebral microbleeds: a review of clinical, genetic, and neuroimaging associations. Front Neurol. (2014) 4:205. 10.3389/fneur.2013.00205 24432010 PMC3881231

[19] van Dijk EJ Prins ND Vrooman HA Hofman A Koudstaal PJ Breteler MM. Progression of cerebral small vessel disease in relation to risk factors and cognitive consequences: rotterdam scan study. Stroke. (2008) 39:2712–9. 10.1161/STROKEAHA.107.513176 18635849

[20] Ungvari Z Tarantini S Kirkpatrick AC Csiszar A Prodan CI. Cerebral microhemorrhages: mechanisms, consequences, and prevention. Am J Physiol Heart Circ Physiol. (2017) 312:H1128–43. 10.1152/ajpheart.00780.2016 28314762 PMC5495931

[21] Nezu T Hosomi N Aoki S Kubo S Araki M Mukai T. Endothelial dysfunction is associated with the severity of cerebral small vessel disease. Hypert Res. (2015) 38:291–7. 10.1038/hr.2015.4 25672660

[22] Pabbidi MR Kuppusamy M Didion SP Sanapureddy P Reed JT Sontakke SP. Sex differences in the vascular function and related mechanisms: role of 17β-estradiol. Am J Physiol Heart Circ Physiol. (2018) 315:H1499–518. 10.1152/ajpheart.00194.2018 30192631

[23] Stanhewicz AE Wenner MM Stachenfeld NS. Sex differences in endothelial function important to vascular health and overall cardiovascular disease risk across the lifespan. Am J Physiol Heart Circ Physiol. (2018) 315:H1569–88. 10.1152/ajpheart.00396.2018 30216121 PMC6734083

[24] Reckelhoff JF Fortepiani LA. Novel mechanisms responsible for postmenopausal hypertension. Hypertension. (2004) 43:918–23. 10.1161/01.HYP.0000124670.03674.15 15023933

[25] Vernooij M Van der Lugt A Ikram MA Wielopolski P Niessen W Hofman A. Prevalence and risk factors of cerebral microbleeds: the rotterdam scan study. Neurology. (2008) 70:1208–14. 10.1212/01.wnl.0000307750.41970.d9 18378884

[26] Tsai H-H Pasi M Tsai L-K Chen Y-F Lee B-C Tang S-C. Microangiopathy underlying mixed-location intracerebral hemorrhages/microbleeds: a PiB-PET study. Neurology. (2019) 92:e774–81. 10.1212/WNL.0000000000006953 30674594 PMC6396971

[27] Pasi M Charidimou A Boulouis G Auriel E Ayres A Schwab KM. Mixed-location cerebral hemorrhage/microbleeds: underlying microangiopathy and recurrence risk. Neurology. (2018) 90:e119–26. 10.1212/WNL.0000000000004797 29247070 PMC5772153

[28] Jung YH Jang H Park SB Choe YS Park Y Kang SH. Strictly lobar microbleeds reflect amyloid angiopathy regardless of cerebral and cerebellar compartments. Stroke. (2020) 51:3600–7. 10.1161/STROKEAHA.119.028487 33198580

[29] Greenberg SM Charidimou A. Diagnosis of cerebral amyloid angiopathy: evolution of the boston criteria. Stroke. (2018) 49:491–7. 10.1161/STROKEAHA.117.016990 29335334 PMC5892842

[30] Maniskas ME Mack AF Morales-Scheihing D Finger C Zhu L Paulter R. Sex differences in a murine model of cerebral amyloid angiopathy. Brain Behav Immun Health. (2021) 14:100260. 10.1016/j.bbih.2021.100260 34589766 PMC8474688

