# Supplementary material for: Sex differences in the progression of cerebral microbleeds in patients with concomitant cerebral small vessel disease
Source: Front Neurol. 2022 Dec 20;13:1054624. doi: 10.3389/fneur.2022.1054624 (PMC9810543; doi:10.3389/fneur.2022.1054624)
Supplement: Supplementary file 1 [file Table_1.DOCX]

**Supplementary Table 1. Comparison between subjects with and without a follow-up magnetic resonance imaging scan**

|  | With a follow-up scan (*n* = 189) | Without a follow-up scan (*n* = 67) | *P-*value |
| --- | --- | --- | --- |
| Age, years | 73.4 (6.8) | 74.8 (6.2) | 0.173 |
| Sex, female | 124 (65.6%) | 39 (58.2%) | 0.303 |
| Hypertension | 156 (82.5%) | 53 (79.1%) | 0.582 |
| Diabetes | 72 (38.1%) | 34 (50.7%) | 0.071 |
| Dyslipidemia | 93 (49.2%) | 33 (49.3%) | 1.000 |
| Current Smoking | 12 (6.3%) | 7 (10.4%) | 0.284 |
| Body mass index, kg/m^2^ | 24.7 (3.1) | 24.5 (3.5) | 0.505 |
| CMBs | *n* = 189 | *n* = 65 |  |
| Presence of CMBs | 135 (71.4%) | 40 (61.5%) | 0.162 |
| Number of CMBs |  |  |  |
| Deep | 1 (0-4) | 1 (0-3) | 0.606 |
| Lobar | 1 (0-2) | 0 (0-3) | 0.678 |
| Total | 2 (0-7) | 2 (0-6) | 0.559 |

The values are presented as number (%), mean (SD), or median (IQR). CMB, cerebral microbleed; SD, standard deviation; IQR, interquartile range.
